# Longitudinal reversals and evolving patterns of the double burden of malnutrition among adults in Southwest China: a population-based cohort study

**DOI:** 10.3389/fnut.2026.1861218

**Published:** 2026-07-08

**Authors:** Hongjiang Long, Yan Sun, Yiya Liu, Peng Luo, Hua Guo

**Affiliations:** 1 Guizhou Center for Disease Control and Prevention, Guiyang, China; 2 Key Laboratory of Environmental Pollution Monitoring and Disease Control, Ministry of Education, School of Public Health, Guizhou Medical University, Guiyang, China

**Keywords:** double burden of malnutrition, generalized estimating equations, joinpoint regression, longitudinal study, nutrition transition

## Abstract

**Background:**

The double burden of malnutrition (DBM), the co-occurrence of micronutrient deficiencies and overweight/obesity, is a rising challenge in transitioning economies. However, longitudinal evidence on its structural evolution and demographic shifts remains limited.

**Objective:**

To analyze the long-term trajectories and socio-metabolic determinants of DBM among adults in Southwest China using three decades of cohort data.

**Methods:**

A total of 10,703 person-wave observations from 3,117 adults (aged ≥18) across 11 rounds of the China Health and Nutrition Survey (the Guizhou cohort, 1991–2023) were analyzed. Nutrient intake was derived from three-day 24-h recalls and household salt/oil weighing. Joinpoint regression characterized temporal trends, and Generalized Estimating Equations (GEE) identified population-averaged associations between DBM and socio-metabolic factors.

**Results:**

DBM prevalence surged from 12.8% (95% CI: 10.8–14.9%) in 1991 to 53.4% (95% CI: 49.8–56.9%) in 2023, with an overall AAPC of 4.97% (95% CI: 4.21–5.74%). Growth significantly decelerated after the 2015 inflection point, shifting from an APC of 5.37% (95% CI: 4.50–6.24%) to 3.79% (95% CI: 1.11–6.55%; *p* = 0.013). Notably, distinct growth rates across subgroups led to a reversal of prevalence patterns by 2023, at which point DBM prevalence in rural areas (53.8%) and among males (57.6%) exceeded that of their urban and female counterparts. GEE models further indicated that while urban residency, female gender, and higher education were historically associated with higher DBM risk, the absence of hypertension (OR: 0.79; 95% CI: 0.71–0.87) and higher riboflavin intake (OR: 0.74; 95% CI: 0.62–0.89) were significantly associated with a lower risk.

**Conclusion:**

The DBM landscape in this regional cohort has undergone a fundamental shift, with the burden shifting toward rural residents and males. Targeted public health strategies should prioritize these emerging high-risk groups, emphasizing dietary improvements such as riboflavin optimization to mitigate the dual burden of adiposity and micronutrient inadequacy.

## 1 Introduction

The coexistence of undernutrition and overnutrition has emerged as a significant global health threat, leading the United Nations to prioritize the eradication of all forms of malnutrition through the Decade of Action on Nutrition initiative (1). Traditionally, public health strategies primarily addressed undernutrition, specifically stunting, wasting, and anemia resulting from food scarcity and socioeconomic deprivation (2). However, rapid socioeconomic shifts and technological progress over the last thirty years have fundamentally altered the global nutritional landscape. While childhood and adult obesity rates have surged dramatically since 1990 (3, 4), nearly two billion people globally continue to suffer from micronutrient deficiencies, a condition often termed hidden hunger (5). This structural shift has given rise to the Double Burden of Malnutrition (DBM), characterized by the simultaneous or sequential occurrence of undernutrition and overnutrition within individuals, households, and populations (6).

The manifestation of DBM occurs across multiple levels of organization and is especially prevalent in low- and middle-income countries (LMICs) where nutritional transitions occur at an accelerated pace. On a regional and national scale, this burden is driven by the interplay between socioeconomic disparities, shifting dietary patterns, and rapid urbanization (7), a trend increasingly observed across LMICs in Africa, Asia, and Latin America (8–10). In these transitioning societies, undernutrition is no longer solely characterized by wasting but is progressively being supplanted by rising adiposity (11, 12). At the individual level, DBM is characterized by the co-occurrence of overweight or obesity with specific forms of undernutrition, such as micronutrient deficiencies or anemia (13). This individual-level burden is often a lifelong trajectory, frequently rooted at the household level where family members experience the “dual dilemma” of early-life undernutrition followed by overnutrition in later years [7]. Collectively, these global patterns across LMICs underscore a typical nutritional transition wherein metabolic risks accumulate rapidly even as traditional nutrient inadequacies remain unresolved (14).

As one of the world’s most populous country experiencing rapid urbanization and shifting dietary landscapes, China has seen a marked escalation in the complexity of its nutritional challenges. National surveillance indicates that DBM prevalence among Chinese children and adolescents has risen by 18.1% since 1991 (15). While adult obesity rates have doubled over the past two decades, with particularly rapid growth among males and rural residents (16), undernutrition persists as a widespread issue. Over 60% of adults do not meet the Estimated Average Requirements (EAR) for essential vitamins and calcium (17). Despite extensive research on malnutrition, most longitudinal studies have focused on pediatric populations or cross-sectional data, leaving a gap in long-term dynamic analyses of DBM among adults. This study utilizes longitudinal data from the China Health and Nutrition Survey (CHNS) in Guizhou Province to examine the spatiotemporal evolution of individual-level DBM from 1991 to 2023. By evaluating long-term trends and disparities across gender and residence, this research aims to identify the underlying dietary and behavior drivers of DBM within the broader context of China’s nutritional transition.

## 2 Methods

### 2.1 Study design and population

This study utilized longitudinal data from 11 rounds of the CHNS conducted at the Guizhou surveillance site between 1991 and 2023(the initial 1989 survey wave was excluded due to the lack of physical examinations). The CHNS is a prospective, dynamic cohort study that employs a multistage, stratified, randomized cluster sampling strategy. This hierarchical approach selected diverse urban and rural communities (neighborhood committees and villages) to represent various socioeconomic levels, with 20 households randomly sampled per community and all family members surveyed. Detailed descriptions of the CHNS methodology have been documented elsewhere (18). Given the three decades of the cohort, a dynamic recruitment strategy was implemented to maintain sample size and population representativeness. Participants or households lost to follow-up—due to migration, mortality, or non-response- were systematically replenished by new participants recruited from the same sampling units who were likely to participate in subsequent survey waves.

**Figure 1:**
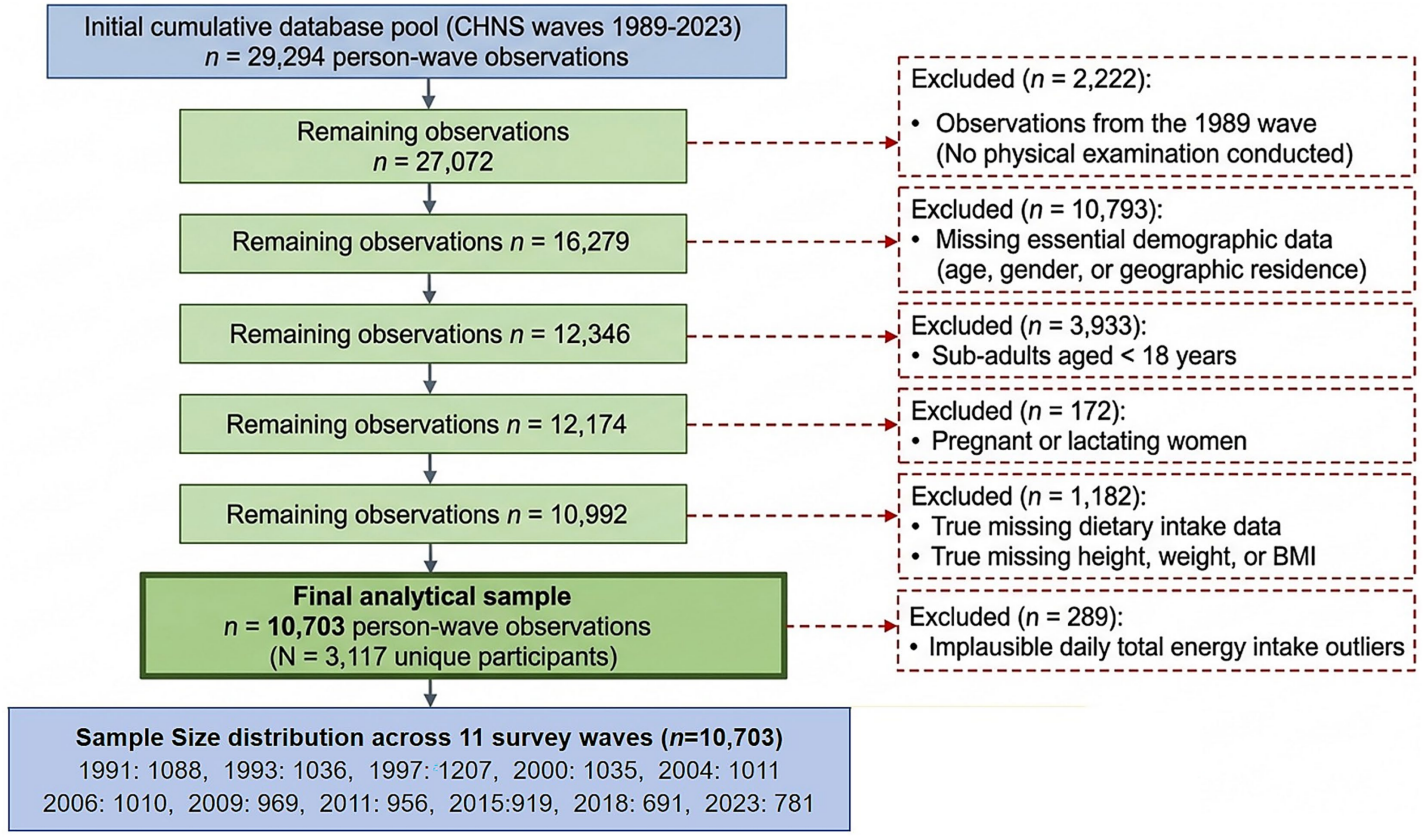
Flowchart of participant selection from the Guizhou surveillance site of the China Health and Nutrition Survey (1991–2023).

The step-by-step screening of participants and their corresponding person-wave observations is detailed in Figure 1. To isolate the target analytic sample, exclusions were applied sequentially. First, observations with missing essential socio-demographic information were removed. Second, the sample was restricted to adults aged > = 18 years, and pregnant or lactating women were excluded to control for physiological confounding. Third, observations with true missing data on dietary intake or anthropometric measurements were eliminated. Finally, observations presenting extreme and biologically implausible daily total energy intakes (> 6,000 kcal/day or < 800 kcal/day for men; > 5,000 kcal/day or < 600 kcal/day for women) in any survey round were discarded to ensure data validity.

Ultimately, the analytical sample comprised 10,703 valid observations clustered within 3,117 unique participants. The cross-sectional observations sample size across individual survey rounds ranged from 1,088 in 1991 to 781 in 2023. The detailed round-specific dynamic compositions and individual attendance frequencies are documented in Supplementary Tables S1, S2, respectively. Repeated measurements from longitudinal historical follow-up accounted for 89.6% of the total observations, with nearly two-thirds (62.3%) of the data contributed by a persistent longitudinal sample who attended 5 or more survey rounds (averaging 7.33 rounds per capita).

All procedures in this study adhered to the National Research Council Ethical Standards, the 1964 Declaration of Helsinki and its later revisions, or similar ethical standards. The study protocol was approved by the Ethical Review Committee of the Institute of Nutrition and Health, CDC (no. 2022-024), and all participants provided written informed consent.

### 2.2 Dietary nutrient intake assessment

Dietary intake data were collected using a 24-h recall method over three consecutive days (two weekdays and one weekend day), combined with the weighing of household seasonings. Individual food consumption was recorded by trained enumerators, while household-level cooking oil and condiment use was measured using precision electronic scales and proportionally allocated to individuals based on their total energy intake. To reflect the dietary profiles of each period, daily energy and nutrient intakes were independently estimated for each survey wave using the relevant versions of the Chinese Food Composition Table (19). These assessments covered macronutrients (carbohydrates, fats, and proteins), vitamins (B1, B2, C, and E), and minerals (calcium, magnesium, iron, zinc, and selenium).

### 2.3 Overweight and obesity assessment

Anthropometric measurements were conducted by medical professionals who had undergone standardized training. Height was measured using a SECA 213 portable stadiometer (accuracy: 0.1 cm), and weight was recorded using a Paradigm TANITA BC601 Body Composition Analyzer (accuracy: 0.1 kg), calibrated prior to each use. BMI was calculated as weight (kg) divided by height squared (m^2^) and classified according to WHO criteria: underweight (<18.5 kg/m^2^), normal weight (18.5–24.9 kg/m^2^), overweight (25.0–29.9 kg/m^2^), and obese (≥30 kg/m^2^).

### 2.4 Definition of intraindividual DBM

DBM at the individual level was defined as the coexistence of overweight/obesity (BMI ≥ 25 kg/m^2^) and micronutrient deficiency during the same survey cycle. Micronutrient deficiency was determined based on the Dietary Nutrient Reference Intake for Chinese Residents (20), with individuals classified as deficient if they consumed below the EAR for at least three micronutrients from the following: vitamin B1, vitamin B2, vitamin C, vitamin E, calcium, magnesium, iron, zinc, and selenium.

### 2.5 Covariate assessment

Demographic variables included gender, age, ethnicity (Han Chinese or minority), residence (urban or rural), and educational level (Middle school and above vs. Elementary school and below). Smoking status was defined as current smoking at the time of each survey round, and alcohol consumption was defined as drinking at least once per week. Hypertension was identified based on self-reported doctor diagnosis, antihypertensive medication use, or three consecutive elevated blood pressure readings (systolic blood pressure ≥140 mmHg or diastolic blood pressure ≥90 mmHg) at rest. Chronic illnesses—including diabetes, fatty liver disease, and stroke, were determined based on self-reported physician diagnosis or prior hospitalization records.

### 2.6 Trend analysis

Temporal trends in DBM prevalence were analyzed using the Cochran-Armitage trend test, while the dynamics of nutrient intake were assessed using the Mann-Kendall trend test for continuous variables. To identify significant inflection points in DBM prevalence, joinpoint regression analysis was employed (Joinpoint Regression Program, version 5.3.0; National Cancer Institute, United States) (21). The analysis progressed from a zero-joinpoint model (linear trend) up to a maximum of four joinpoints. The annual percentage change (APC) for each identified segment and the average annual percentage change (AAPC) for the entire study period were estimated. Subgroup analyses were further conducted by gender and urban/rural residence, with corresponding 95% confidence intervals (CIs) calculated to evaluate the stability of these trends (22).

### 2.7 Generalized estimating equations

To examine the longitudinal associations between covariates and DBM within the repeated-measures cohort, Generalized Estimating Equations (GEE) were employed with a logit link function (binomial family). A first-order autoregressive (AR1) working correlation structure was specified to account for within-individual correlation and its potential decay over time. The selection of covariates was determined based on well-established evidence linking socio-demographic transitions, lifestyle behaviors, and metabolic burdens to individual-level nutritional shifting in prior longitudinal cohort studies (23–25). To rigorously evaluate the independent effects of various factors and ensure the robustness of the findings, three progressive adjustment models were constructed:

- Model 1: Included core socio-demographic indicators, specifically survey wave (year), age, gender, residence (urban/rural), and educational attainment.
- Model 2: Built upon Model 1 by incorporating health-related and behavioral indicators, including smoking status, alcohol consumption, history of hypertension, and other chronic diseases.
- Model 3 (Fully Adjusted Model): Further incorporated dietary nutrient intakes into Model 2. To capture the independent biological effects of individual nutrients while addressing the inherent multidimensional correlation in dietary data, a multi-nutrient framework was implemented by simultaneously introducing all candidate nutrients, total energy intake, and macronutrient energy ratios into a preliminary multivariable GEE model. To minimize Type II errors caused by variance inflation under collinearity, nutrients with *p* < 0.1 in this fully adjusted environment were retained for the final model (Supplementary Table S3). Subsequently, Variance Inflation Factors (VIF) were evaluated to ensure the absence of severe multicollinearity (VIF < 5) among the remaining variables. Finally, model parsimony and goodness-of-fit were confirmed using the Quasi-likelihood under the Independence Model Criterion (QIC), where lower values indicate superior model fit.

Furthermore, the heterogeneity of DBM prevalence trends across subgroups was examined by introducing interaction terms for survey wave by residence and survey wave by gender into Model 3. For interactions reaching statistical significance (*p* < 0.05), marginal predicted probabilities for each subgroup were calculated.

### 2.8 Statistical analysis

Data cleaning and analysis were performed using SAS 9.4 and R 4.4.2. Baseline characteristics and their longitudinal trends across survey waves were summarized as medians (interquartile ranges, IQRs) for continuous variables and as frequencies (percentages) for categorical variables. Categorical covariates were dummy-coded for regression analysis, with reference categories selected based on population distribution and clinical relevance. All *p*-values were two-sided, and *p* < 0.05 was considered statistically significant.

## 3 Results

### 3.1 Demographic characteristics and temporal trends

Between 1991 and 2023, the median age of the study population increased significantly from 40.1 years (IQR: 26.8–53.5) in 1991 to 54.0 years (IQR: 44.0–68.0) in 2023 (P for trend <0.001). The proportion of individuals aged 65 years and older rose markedly from 8.9 to 31.2% over the study period. Meanwhile, the proportion of male participants declined from 47.7 to 41.1% (P for trend = 0.002), while the distribution of urban residents remained stable (34.0% in 1991 vs. 32.9% in 2023, P for trend = 0.80). Educational level showed a significant improvement, with the proportion of individuals attaining middle school education or higher increasing from 35.4 to 58.1% (P for trend <0.001). The prevalence of hypertension and other chronic diseases also rose substantially, from 15.5% (95% CI, 14.9–16.1%) and 0.00% in 1991 to 51.1% (95% CI, 50.06–52.14%) and 3.8% (95% CI, 3.41–4.19%) in 2023, respectively (both P for trend <0.001). Further details on nutrient intake and additional demographic characteristics are presented in Table 1.

**Table 1:** Trends in population demographics and nutritional status (1991–2023).

Characteristics	1991 (*n* = 1,088)	1993 (*n* = 1,036)	1997 (*n* = 1,207)	2000 (*n* = 1,035)	2004 (*n* = 1,011)	2006 (*n* = 1,010)	200 9 (*n* = 969)	2011 (*n* = 956)	2015 (*n* = 919)	2018 (*n* = 691)	2023 (*n* = 781)	*p* value	*Z*	*p* for trend
Age	40.1 (26.8,53.5)	41.3 (27.8,54.3)	43.4 (28.9,55.8)	46 (32,57.7)	49.9 (35.9,60.2)	51.6 (38.1,61.9)	54.2 (40.5,64.2)	55.1 (40.6,65)	51.5 (40,65.3)	54.3 (43.3,67.6)	54 (44,68)	<.0.001	27.68	<0.01
Gender (%)												0.04	−3.21	0.002
Male	519 (47.7)	484 (46.7)	581 (48.1)	486 (47.0)	482 (47.7)	476 (47.1)	463 (47.8)	453 (47.4)	411 (44.7)	292 (42.3)	321 (41.1)			
Female	569 (52.3)	5,552 (53.3)	626 (51.9)	549 (53.0)	529 (52.3)	534 (52.9)	506 (52.2)	503 (52.6)	508 (55.3)	399 (57.4)	450 (58.9)			
Living area												0.49	1.2	0.8
Urban	370 (34.0)	310 (29.9)	374 (31.0)	325 (31.4)	305 (30.2)	319 (31.6)	289 (29.8)	297 (31.1)	278 (30.1)	232 (33.6)	257 (32.9)			
Rural	718 (66.0)	726 (70.1)	833 (69.0)	710 (68.6)	706 (69.8)	691 (68.4)	680 (70.2)	659 (68.9)	641 (69.8)	459 (66.4)	524 (67.1)			
Ethnicity (%)												0.03	−1.56	0.12
Chinese (Han)	450 (41.4)	499 (48.2)	539 (44.7)	451 (43.6)	448 (44.3)	467 (46.2)	430 (44.4)	448 (46.9)	430 (46.8)	341 (49.4)	253 (32.4)			
Minority	638 (58.6)	537 (51.8)	668 (55.3)	584 (56.4)	563 (55.7)	543 (53.8)	539 (55.6)	508 (53.1)	489 (53.2)	350 (50.7)	528 (67.6)			
Education level (%)												<.0.001	−23.28	<0.01
Elementary school and below	703 (64.6)	647 (62.5)	676 (56.0)	553 (53.4)	597 (59.0)	564 (55.8)	529 (54.6)	509 (53.2)	462 (50.3)	366 (53.0)	327 (41.9)			
Middle school and above	385 (35.4)	389 (37.6)	531 (44.0)	482 (46.6)	414 (41.0)	446 (44.2)	440 (45.4)	447 (46.8)	457 (49.7)	325 (47.0)	454 (58.1)			
Smoking status (%)												<.0.001	8.28	<0.01
No	702 (64.5)	673 (65.0)	769 (63.7)	692 (67.2)	679 (67.2)	690 (68.3)	633 (65.3)	632 (64.5)	693 (75.4)	517 (74.8)	607 (77.7)			
Yes	386 (35.5)	363 (35.0)	438 (36.3)	332 (32.8)	332 (32.8)	330 (31.7)	336 (34.7)	324 (33.9)	226 (24.6)	174 (25.2)	174 (22.3)			
Alcohol (%)												<.0.001	—8.17	<0.01
No	643 (59.1)	623 (60.1)	728 (60.3)	639 (61.7)	664 (65.7)	713 (70.6)	634 (65.4)	612 (64.0)	661 (71.9)	505 (73.1)	526 (67.4)			
Yes	445 (40.9)	413 (39.9)	479 (39.7)	396 (28.3)	347 (34.3)	297 (29.4)	335 (34.6)	344 (36.0)	258 (28.1)	186 (26.9)	255 (32.6)			
Hypertension (%)												<.0.001	23.98	<0.01
Yes	169 (15.5)	144 (13.9)	233 (19.3)	233 (22.5)	242 (23.9)	284 (28.1)	339 (35.0)	329 (34.4)	315 (34.3)	301 (43.6)	399 (51.1)			
No	919 (84.5)	892 (86.1)	974 (80.7)	802 (77.5)	769 (76.1)	726 (71.9)	630 (65.0)	627 (65.6)	604 (65.7)	390 (56.4)	382 (48.9)			
Chronic disease (%)												<0.001		
Yes	0 (0)	0 (0)	8 (0.7)	14 (1.3)	9 (0.9)	20 (2.0)	25 (2.6)	41 (4.3)	20 (2.2)	25 (3.6)	30 (3.8)		−10.00	<0.01
No	1,088 (100)	1,036 (100)	1,199 (99.3)	1,021 (98.7)	1,002 (99.1)	990 (98.0)	944 (97.4)	915 (95.7)	899 (97.8)	666 (96.4)	751 (96.2)			
Kcal (g/d)	2663.8 (2265.2,3141.4)	2559.58 (2137.3,3020.3)	2641.0 (2148.5,3164.2)	2281.4 (1840.1,2783.1)	2443.1 (1992.8,2930.4)	2368.5 (1895.6,2,928)	2071.1 (1669.7,2608.6)	1849.2 (1509.9,2365.8)	1836.2 (1456.2,2233.4)	1838.5 (1477.3,2305.2)	1739.5 (1376.9,2298.8)	<.0.001	−42.16	<.0.001
Fat (g/d)	59.9 (39.9,92.1)	59.1 (33.1,88.7)	65.4 (41.5,96.1)	66.4 (41.6,99.9)	70.4 (45.2,102.4)	72.0 (47.2,105.0)	70.3 (47.5,98.2)	72.4 (52.5,103.6)	78.1 (56.3,103.3)	77.9 (54.9,108.7)	81.9 (58.3,123.8)	<.0.001	14.72	<.0.001
Carbohydrate (g/d)	422.1 (335.3,512.9)	413.5 (327.5,497.2)	406.3 (326.3,505.3)	329.5 (251.7,415.1)	360.1 (292.8,442.4)	325.7 (240.1,409)	273.9 (219.7,344.9)	234.9 (189,283.6)	213 (167.2,267.8)	221.3 (173.1,275.8)	187 (141.4,249.4)	<.0.001	−62.06	<.0.001
Protein (g/d)	78.6 (64.7,94.8)	76.1 (61.6,92.2)	73.4 (59,89.8)	62.5 (51.1,77.2)	65 (52.9,82)	61.8 (47.6,77.3)	61.1 (48.4,77.9)	53.6 (42.7,66.5)	56.5 (43.1,71.9)	57 (45.4,71.3)	49.9 (39.6,65.6)	<.0.001	−38.04	<.0.001
Fibre (g/d)	14.4 (10.5,20.2)	13.1 (9.9,18.1)	11.4 (7.9,18.1)	9.8 (6.7,14.5)	10.6 (7.8,14.4)	10.3 (7.5,14.3)	10 (7.1,13.7)	10.2 (7.4,14.8)	11 (7.9,15.3)	11.2 (7.4,16.4)	8.5 (5.6,13)	<.0.001	−19.14	<.0.001
FER (%)	21.6 (14.5,29.9)	21.6 (11.9,31.6)	23.6 (15.6,32.5)	27.1 (18.3,38.2)	26.9 (18.4,36.1)	28.6 (20.1,38.6)	30.5 (23.1,38.2)	36.3 (28.6,43.5)	39.2 (31.5,47.2)	38.7 (30.9,47.4)	44.2 (35.9,53.5)	<.0.001	46.76	<.0.001
CER (%)	64.7 (55.2,72.3)	64.6 (54.9,74.3)	63.9 (54.8,72.4)	59.8 (48.7,69.8)	61.2 (51.5,70.1)	56.6 (44.6,66.3)	54.5 (44.9,63.2)	51 (44,58.3)	48.1 (38.7,55.3)	48.4 (39.4,56.6)	43.3 (34.2,51.6)	<.0.001	−45.09	<.0.001
PER (%)	11.9 (10.6,13.2)	11.9 (10.7,13.2)	11.2 (9.9,12.5)	10.9 (9.7,12.8)	10.6 (9.3,12.8)	10.4 (9,11.9)	11.5 (10.2,13.5)	11.4 (9.8,13.3)	12.5 (10.3,15)	12.3 (10.3,14.5)	11.8 (9.7,14.2)	0.09	1.90	0.46
Vitamin B1 (mg/d)	1.1 (0.9,1.3)	1.1 (0.9,1.4)	1.1 (0.9,1.4)	1 (0.8,1.2)	1 (0.8,1.2)	1 (0.7,1.2)	0.9 (0.7,1.2)	0.8 (0.6,1)	0.8 (0.6,1.1)	0.8 (0.6,1)	0.7 (0.5,1)	<.0.001	−36.42	<.0.001
Vitamin B2 (mg/d)	0.7 (0.6,1)	0.7 (0.5,0.9)	0.7 (0.5,0.9)	0.7 (0.5,0.9)	0.7 (0.5,0.9)	0.7 (0.5,0.9)	0.7 (0.5,0.9)	0.6 (0.5,0.8)	0.7 (0.5,0.9)	0.7 (0.5,0.9)	0.6 (0.5,0.8)	<.0.001	−9.85	<.0.001
Vitamin C (mg/d)	85.1 (58.3,125)	94.5 (65.1,139.6)	69.8 (45.7,105.2)	88 (59.8,129.5)	77.2 (51.1,117.7)	72 (46.2,105.8)	70.9 (48.7,102.4)	70.4 (45.8,100.9)	70.8 (45.7,113.3)	61.8 (40.7,96.7)	42.1 (20.6,76.4)	<.0.001	−22.23	<.0.001
Vitamin E (mg/IU)	15.9 (9.2,25.1)	16 (8.9,24.7)	19.4 (11.3,31.1)	16.7 (9.8,27.6)	22 (13.2,33.6)	19.3 (11.9,32.2)	18.7 (10.5,30.3)	22 (13.3,34.5)	19.8 (12.7,30.1)	19.5 (10.8,31.3)	20.9 (12.3,32.7)	<.0.001	10.14	<.0.001
Ca (mg/d)	323 (237.5,444.1)	311 (231.4,403.7)	361.9 (259.1,494.9)	357.2 (258,494.3)	357 (272.3,486.3)	333.2 (234.8,447.3)	372.4 (268.3,495.4)	318.7 (229.1,436)	315.2 (209.7,418.9)	305 (209.9,401.1)	261 (179.9,368.7)	<.0.001	−9.24	<.0.001
Mg (mg/d)	397.4 (325.6,474.2)	372.6 (302.1,447.1)	356.4 (282.2,453.4)	292.5 (239.2,373.4)	312.5 (255.7,386.4)	290.7 (223.6,363.6)	281.8 (219.7,358.5)	252 (201.3,310.9)	231.8 (181.6,293.8)	240.4 (186,299.8)	200 (152.5,264.9)	<.0.001	−49.58	<.0.001
Fe (mg/d)	19.3 (14.8,26.1)	17.4 (13.4,22.8)	29.2 (22.4,37.4)	24.7 (19.6,31.4)	22.4 (18.4,27.4)	21.9 (17.2,28.1)	21.1 (16.5,26.8)	20.2 (16.6,24.9)	19.2 (15.4,24.6)	20.1 (15.7,26.3)	18 (13.7,24.4)	<.0.001	−10.06	<.0.001
Zn (mg/d)	11.9 (10,14.8)	11.7 (9.6,13.8)	13.2 (10.7,15.7)	11.4 (9.2,14)	12.1 (9.9,14.5)	11.5 (9,14.4)	11 (8.7,13.5)	10.1 (8.3,12.5)	10.2 (7.7,13.3)	10.4 (8,12.9)	8.9 (6.6,11.8)	<.0.001	−24.99	<.0.001
Se (μg/d)	32.7 (25,42.4)	28 (19.8,38.3)	33.1 (25.3,45.1)	29.6 (22,41.7)	34.9 (25.4,45.9)	33.9 (25.2,44.7)	33.9 (25.5,44.6)	31.9 (23.8,41.6)	35.1 (25.8,46.7)	37.1 (27.9,48)	33.3 (24.3,45.6)	<.0.001	6.29	<.0.001
Cholesterol (mg/d)	88.7 (30,177.6)	73.9 (14.6,164.7)	86.2 (31.6,181.4)	104.5 (42.2,230.5)	98.5 (33.2,238.1)	131.1 (67.5,250)	160 (79.7,296)	153.7 (74.1,290.1)	212.6 (109.1,312)	194.1 (96,303.7)	209.2 (120,356.2)	<.0.001	28.42	<.0.001
BMI	20.9 (19.5,22.5)	20.8 (19.5,22.5)	20.9 (19.4,22.9)	21.3 (19.6,23.5)	21.6 (19.8,23.8)	21.9 (19.9,24)	22.3 (20.2,24.7)	22.2 (20.2,24.9)	23.3 (21,25.8)	23.9 (21.3,26.4)	24.4 (22.2,27)	<.0.001	31.29	<.0.001
Emaciation	116 (10.7)	118 (11.4)	165 (13.7)	115 (11.1)	101 (10.0)	85 (8.4)	79 (8.2)	94 (9.8)	70 (7.6)	37 (5.6)	29 (3.7)			
Normal	829 (76.2)	780 (75.3)	861 (71.3)	712 (68.8)	674 (66.7)	668 (66.1)	584 (60.3)	551 (57.6)	457 (49.7)	315 (45.6)	323 (41.4)			
Overweight	121 (11.1)	12 (11.6)	154 (12.8)	171 (16.5)	194 (19.2)	201 (19.9)	223 (24.1)	241 (25.2)	286 (31.1)	248 (35.9)	290 (37.1)			
Obese	22 (2.0)	18 (1.7)	27 (2.2)	37 (3.6)	42 (4.2)	56 (5.5)	73 (7.5)	70 (7.3)	106 (11.5)	91 (13.2)	139 (17.8)			
Micronutrient Deficiency (%)												0.09	0.14	0.88
Yes	1,052 (96.7)	1,010 (97.5)	1,137 (94.2)	1,005 (97.1)	944 (93.4)	966 (95.6)	916 (94.5)	925 (96.8)	877 (95.4)	666 (96.4)	759 (97.2)			
No	36 (3.31)	26 (2.5)	70 (5.8)	30 (2.9)	67 (6.6)	44 (4.4)	53 (5.5)	31 (3.2)	42 (4.6)	25 (3.6)	22 (2.8)			
DBM (%)												<.0.001	29.08	<.0.001
Yes	139 (12.8)	133 (12.8)	170 (14.1)	200 (19.3)	221 (21.9)	237 (23.5)	290 (29.9)	299 (31.3)	373 (40.6)	324 (46.9)	417 (53.4)			
No	949 (87.2)	903 (87.2)	1,037 (85.9)	835 (80.7)	790 (78.1)	773 (76.5)	679 (70.1)	657 (68.7)	546 (59.4)	367 (53.1)	364 (46.6)			

**Figure 2:**
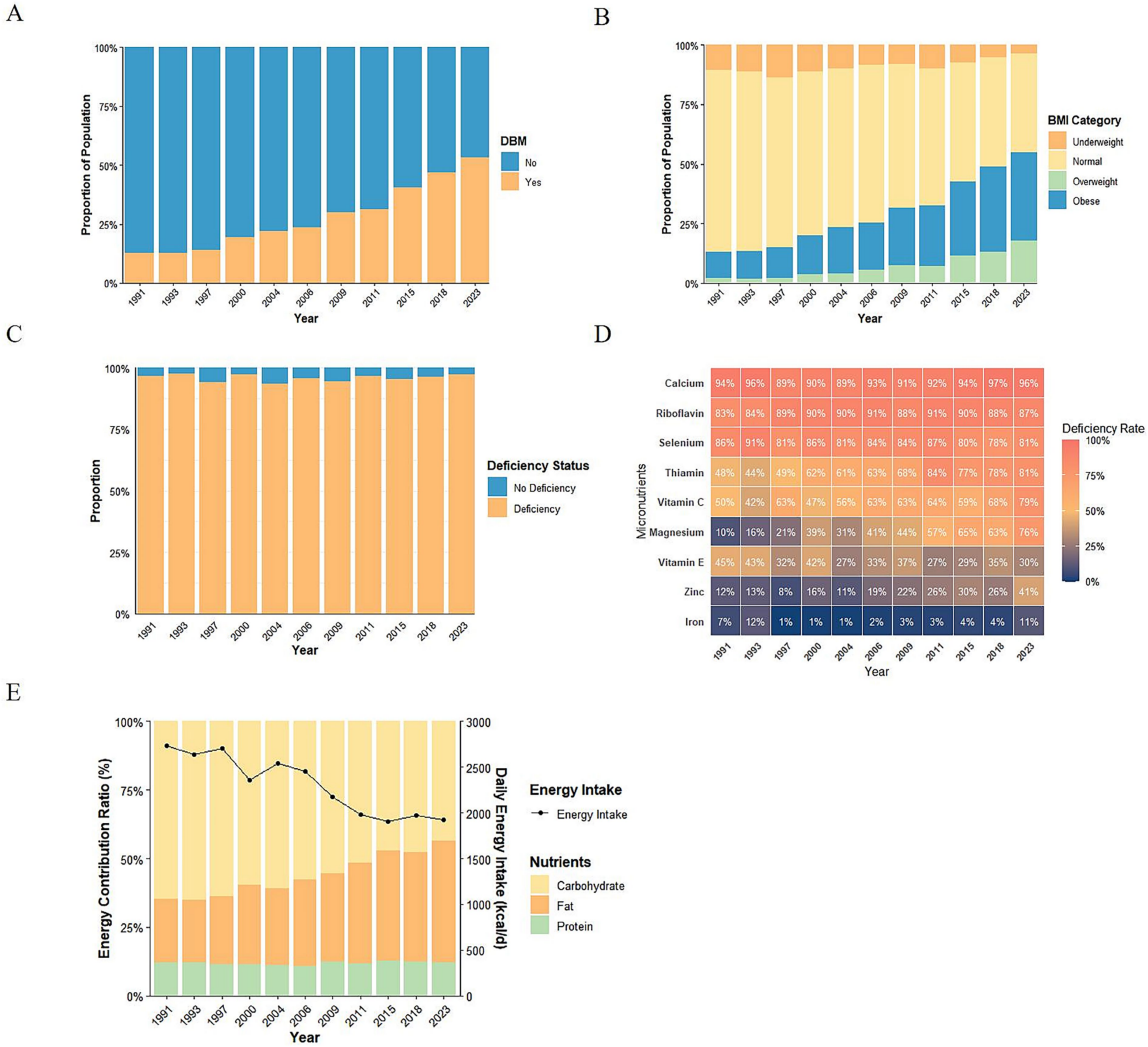
Longitudinal trends in DBM prevalence, BMI distribution, and nutritional profiles from 1991 to 2023. **(A)** Trends in the prevalence of individual-level DBM. **(B)** Distribution shifts in BMI classifications (underweight, normal weight, and overweight/obesity). **(C)** Total proportion of participants with micronutrient deficiencies. **(D)** Specific trends in individual micronutrient deficiency rates. **(E)** Temporal dynamics of macronutrient energy contributions and mean daily total energy intake.

Over the 32-year study period, the prevalence of intra-individual DBM increased significantly from 12.8% (95% CI: 10.8–14.9%) in 1991 to 53.4% (95% CI: 49.8–56.9%) in 2023 (P for trend <0.001) (Figure 2A). A gradual decline was observed in the proportion of individuals categorized as underweight or within the normal BMI range (10.7 to 3.7% and 76.2 to 45.6%, respectively), while the prevalence of overweight and obesity more than tripled (13.1% vs. 54.9%). The median BMI increased from 20.9 kg/m^2^ in 1991 to 24.4 kg/m^2^ in 2023 (P for trend <0.001), with obesity rates rising from 2.0% (95% CI: 1.77–2.23%) to 17.8% (95% CI: 17.01–18.59%) over the same period (Figure 2B). Regarding micronutrient deficiencies, the proportion of individuals with inadequate intake of at least three out of nine key micronutrients (i.e., below the EAR) remained consistently high (>90%) across survey years, with no clear increasing or decreasing trend (Figure 2C). Among the micronutrients assessed, calcium, vitamin B2, and selenium exhibited the highest deficiency rates, each exceeding 80% throughout the study period. Deficiency rates for vitamin B1, vitamin C, magnesium, and zinc showed an increasing trend (P for trend <0.001), while vitamin E was the only micronutrient for which deficiency rates declined over time (Figure 2D). A structural shift in dietary intake patterns was also observed. Total energy intake declined by 34.7% over the study period (from 2663.8 kcal/day in 1991 to 1739.5 kcal/day in 2023), accompanied by a 23% increase in fat intake. Consequently, the proportion of total energy derived from fat nearly doubled from 21.6 to 44.2%, while the carbohydrate energy ratio decreased from 64.7 to 43.3% (P for trend <0.001). In contrast, no significant trend was observed in the contribution of protein to total energy intake (Figure 2E).

### 3.2 Spatiotemporal trends and subgroup reversals in DBM prevalence

**Figure 3:**
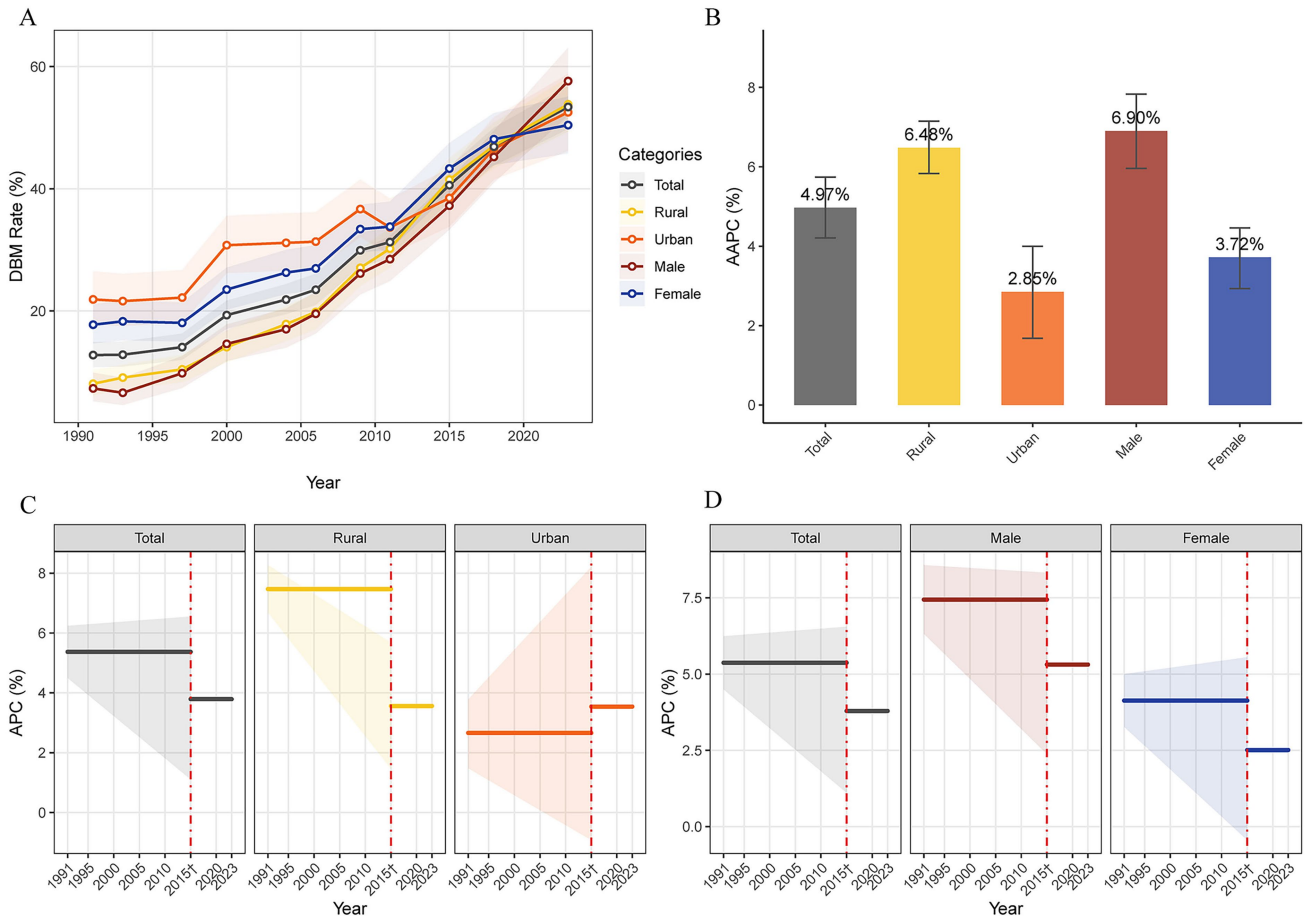
Temporal trends and joinpoint regression trajectories of DBM prevalence stratified by residence and gender. **(A)** Longitudinal trends in DBM prevalence by residence (urban vs. rural) and gender (male vs. female). **(B)** Average Annual Percent Change (AAPC) for each subgroup over the 32-year study period. **(C)** Joinpoint regression segments and inflection points for urban and rural areas. **(D)** Joinpoint regression segments and inflection points for males and females. Error bars represent 95% confidence intervals.

Joinpoint regression analysis demonstrated a significant increase in DBM prevalence among adults from 1991 to 2023, rising from 12.8% (95% CI: 10.8–14.9%) to 53.4% (95% CI: 49.8–56.9%), with an overall average annual percentage change (AAPC) of 4.97% (95% CI: 4.21–5.74%) (Figures 3A,B). A distinct inflection point was identified in 2015, marking a shift from a period of rapid growth before 2015 (APC = 5.37, 95% CI: 4.50–6.24%) to a significantly slower increase thereafter (APC = 3.79, 95% CI: 1.11–6.55%; *p* = 0.013) (Table 2).

**Table 2:** Segmented trends and annual percentage changes in DBM prevalence.

Categories	Period	APC (95% CI)	*p* value	AAPC (95% CI)	*p* value
Total	1991–2015	5.37 (4.50–6.24)	< 0.001	4.97 (4.21–5.74)	< 0.001
	2015–2023	3.79 (1.11–6.55)	0.013		
Rural	1991–2015	7.47 (6.69–8.27)	< 0.001	6.48 (5.83–7.15)	< 0.001
	2015–2023	3.56 (1.47–5.68)	0.006		
Urban	1991–2015	2.66 (1.48–3.78)	0.001	2.85 (1.68–4.00)	< 0.001
	2015–2023	3.54 (−0.92–8.21)	0.102		
Male	1991–2015	7.44 (6.32–8.57)	< 0.001	6.90 (5.96–7.83)	< 0.001
	2015–2023	5.31 (2.38–8.32)	0.004		
Female	1991–2015	4.13 (3.27–5.00)	< 0.001	3.72 (2.93–4.46)	< 0.001
	2015–2023	2.51 (−0.44–5.55)	0.083		

The optimal number of joinpoints was determined using a permutation test (maximum of 4 joinpoints allowed).

However, notable heterogeneity was observed across subgroups. Stratified analyses by urban and rural residence revealed that, at baseline (1991), DBM prevalence was substantially higher in urban areas compared to rural areas (21.9, 95% CI: 17.8–26.5% vs. 8.08, 95% CI: 6.19–10.3%). However, by 2023, this pattern had reversed, with rural prevalence surpassing urban prevalence (53.8, 49.4–58.2% vs. 52.5, 46.2–58.8%). This reversal was driven by a significantly faster increase in DBM prevalence in rural areas between 1991 and 2015 (APC = 7.47, 95% CI: 6.69–8.27%), which was nearly 2.8 times higher than the urban growth rate over the same period (APC = 2.66, 1.5–3.8%). Although the rural growth rate declined to 3.56% (95% CI: 1.47–5.68%) after 2015, it remained higher than the non-significant urban trend (APC = 3.54, 95% CI: −0.92–8.21%; *p* = 0.102), resulting in a significant disparity in overall AAPC between rural and urban areas (6.48, 95% CI: 5.83–7.15% vs. 2.85, 95% CI: 1.68–4.00%; *p* < 0.001).

Gender-based analyses further highlighted substantial differences. At baseline, DBM prevalence was significantly lower in males than in females (7.32, 95% CI: 5.23–9.91% vs. 17.8, 95% CI: 14.7–21.1%). However, by 2023, this trend had reversed, with male prevalence surpassing that of females (57.6, 52.0–63.1% vs. 50.4, 45.8–55.1%). Segmented modeling revealed that males exhibited a persistently high growth rate up to 2015 (APC = 7.44, 95% CI: 6.32–8.57%), followed by a continued significant annual increase of 5.31% (95% CI: 2.38–8.32%; *p* = 0.004) after 2015. In contrast, the growth rate among females between 1991 and 2015 was 4.13% (95% CI: 3.27–5.00%), which declined to a non-significant 2.51% (95% CI: −0.44–5.55%; *p* = 0.083) after 2015. Ultimately, the overall AAPC for males (6.90, 95% CI: 5.96–7.83%) was nearly twofold that of females (3.72, 2.93–4.46%), reflecting a markedly steeper accumulation of DBM risk among men (Figures 3C,D).

### 3.3 Longitudinal predictors and subgroup interactions of DBM

**Figure 4:**
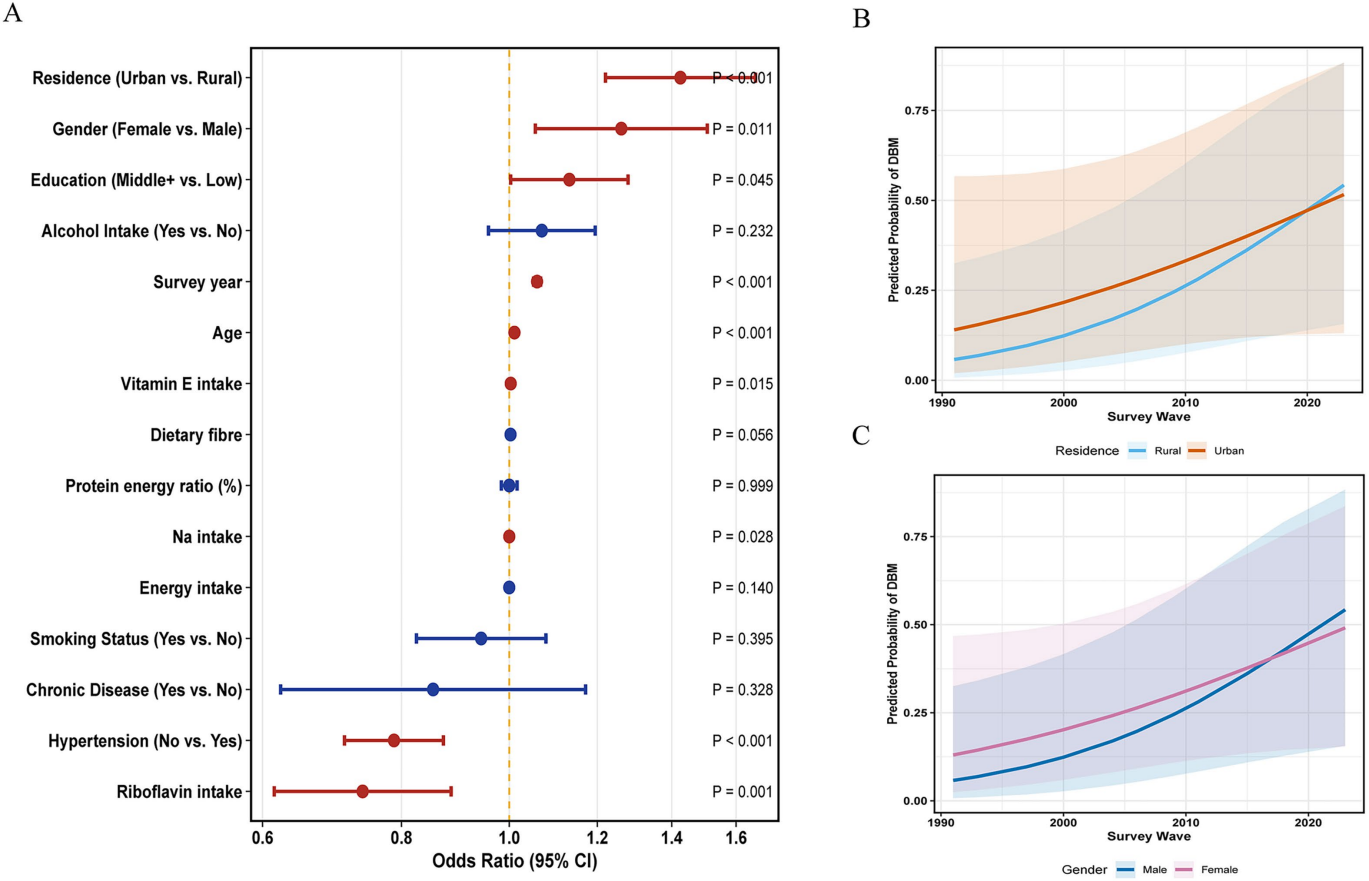
Longitudinal predictors and subgroup interaction patterns for DBM risk based on generalized estimating equations. **(A)** Forest plot showing adjusted odds ratios (ORs) and 95% confidence intervals for independent predictors of DBM. **(B)** Interaction effect of survey wave and residence on the predicted probability of DBM. **(C)** Interaction effect of survey wave and gender on the predicted probability of DBM.

To assess the longitudinal determinants of DBM, multivariable GEE models were constructed as detailed in Table 3. Model 3, which incorporated socio-demographic factors, behavioral and health factors, and dietary factors screened via QIC (*p* < 0.1), demonstrated the most robust fit (QIC = 10,962). Across the 32-year study period, each survey wave was associated with a 5.9% increase in DBM risk (OR = 1.059, 95% CI: 1.052–1.067, *p* < 0.001) (Figure 4A).

**Table 3:** Results of GEE models for associated factors of DBM.

Characteristics	Model 1		Model 2		Model 3	
	OR (95% CI)	*p*	OR (95% CI)	*p*	OR (95% CI)	*p*
Socio-demographic						
Survey year (per wave)	1.066 (1.058, 1.073)	<0.001	1.062 (1.055, 1.070)	<0.001	1.059 (1.052, 1.067)	<0.001
Age	1.013 (1.009, 1.017)	<0.001	1.012 (1.008, 1.016)	<0.001	1.011 (1.007, 1.015)	<0.001
Gender (female vs. male)	1.302 (1.118, 1.517)	0.001	1.327 (1.113, 1.583)	0.002	1.261 (1.056, 1.507)	0.011
Residence (urban vs. rural)	1.478 (1.274, 1.716)	<0.001	1.430 (1.229, 1.664)	<0.001	1.426 (1.221, 1.665)	<0.001
Education (middle+ vs. low)	1.136 (1.007, 1.281)	0.038	1.133 (1.003, 1.280)	0.044	1.133 (1.003, 1.280)	0.045
Behavioral and Health						
Alcohol intake (yes vs. no)	—	—	1.059 (0.948, 1.184)	0.31	1.070 (0.957, 1.196)	0.232
Smoking status (yes vs. no)	—	—	0.940 (0.821, 1.077)	0.373	0.943 (0.825, 1.079)	0.395
Hypertension (no vs. yes)	—	—	0.789 (0.712, 0.874)	<0.001	0.787 (0.711, 0.872)	<0.001
Chronic disease (yes vs. no)	—	—	0.901 (0.661, 1.228)	0.51	0.854 (0.622, 1.171)	0.328
Dietary Intake						
Total ENERGY	—	—	—	—	1.000 (1.000, 1.000)	0.14
Protein energy	—	—	—	—	0.999 (0.983, 1.016)	0.999
Dietary fiber	—	—	—	—	1.003 (1.000, 1.005)	0.056
Na	—	—	—	—	1.000 (1.000, 1.000)	0.028
Vitamin E	—	—	—	—	1.003 (1.001, 1.005)	0.015
Riboflavin	—	—	—	—	0.738 (0.615, 0.886)	0.001
Interactions						
Wave × Residence (urban vs. rural)	—	—	—	—	0.967 (0.954, 0.980)	<0.001
Wave × Gender (female vs. male)	—	—	—	—	0.966 (0.954, 0.979)	<0.001
QIC	11,281		11,147		10,962	

Regarding socio-demographic and health factors, urban residence (OR = 1.426, 95% CI: 1.221–1.665) and female (OR = 1.261, 95% CI: 1.056–1.507) were major independent risk factors. Higher educational attainment was also associated with an increased risk of DBM (OR = 1.133, 95% CI: 1.003–1.280, *p* = 0.045). In terms of clinical and dietary risks, the absence of hypertension (OR = 0.787, 95% CI: 0.711–0.872) and higher riboflavin intake (OR = 0.738, 95% CI: 0.615–0.886) significantly reduced the risk of DBM, while higher sodium intake remained a statistically significant risk factor. Other dietary components, including Vitamin E and dietary fibre, showed subtle positive associations, whereas other factors did not reach statistical significance.

Significant interactions were observed between survey wave and residence (*p* < 0.001), as well as between wave and gender (*p* < 0.001). While urban and female participants faced a higher cumulative risk, the negative interaction coefficients (OR < 1) indicate a shifting landscape. As illustrated by the marginal predicted probability plots (Figures 4B,C), the rate of DBM expansion in rural areas and among males was significantly steeper than in their urban and female counterparts, providing evidence for the trend reversals observed in the joinpoint analysis.

## 4 Discussion

This study analyzes the 32-year dynamic evolution of the DBM among adults in Southwest China, a period marked by socioeconomic restructuring and an accelerated nutritional transition. Our findings demonstrate that DBM has transitioned from a sporadic concern primarily involving traditional undernutrition into a dominant public health problem. This trajectory is characterized by a fundamental structural shift within the DBM complex: while conventional indicators of undernutrition, such as wasting, have regressed to marginal levels, they have been rapidly supplanted by a surge in overweight and obesity prevalence alongside persistent micronutrient inadequacies.

The utilization of a dual-modeling approach provides a nuanced perspective on this transition by capturing both long-term accumulation and temporal shifts. While the GEE models identify urban residency and female gender as the primary factors associated with the cumulative historical burden of DBM, the joinpoint regression analysis uncovers a critical temporal inflection point around 2015. This analysis highlights a notable “catch-up” effect in rural areas and among males, where DBM growth rates have significantly outpaced those of their counterparts, culminating in a complete shift of traditional risk disparities by 2023. These results indicate that the epidemiological landscape of DBM is being reshaped, necessitating a strategic reassessment of public health priorities.

This observed trend aligns with broader national shifts in dietary structures and lifestyle behaviors. Over the past three decades, the concurrent trends of declining total energy intake and rising overweight and obesity rates observed in our regional cohort parallel the broader patterns documented across China (26). National surveillance data suggest that this phenomenon is closely linked to reduced physical activity resulting from rapid urbanization and changes in transportation modes (27, 28). Simultaneously, a shift toward diets with higher fat-to-energy ratios and decreased carbohydrate contributions has been shown to significantly elevate adiposity risks (29, 30). Crucially, these changes have not resolved persistent micronutrient inadequacies. The prevalence of inadequate intake for essential nutrients like calcium and vitamin B2 remains high and demonstrates an upward trajectory (31). The inverse association between BMI and micronutrient intake further complicates this landscape, as overweight individuals often exhibit higher susceptibility to nutrient deficiencies due to poor dietary quality (32–34).

The observed crossover and reversal in DBM prevalence across urban–rural and gender subgroups reflect a profound evolution in the epidemiological risk landscape. While GEE models characterize the population-averaged effects, the significant interactions between survey waves and both residence and gender provide a robust statistical basis for this spatiotemporal heterogeneity. This indicates that DBM growth trajectories are not synchronized across subpopulations, with the growth momentum increasingly decoupling from traditional high-risk groups. The accelerated growth rate observed in rural areas appears to be linked to a synergistic effect of declining physical expenditure and shifting dietary compositions. This pattern aligns with findings from the United States, where overweight/obesity prevalence and growth rates are higher in non-metropolitan areas compared to urban centers (35). The increasing penetration of high-calorie processed foods in rural regions, mechanization-induced declines in physical activity, and a lagged yet ongoing shift toward Westernized diets may have collectively coincided with this trend (28, 36, 37). In this context, rural populations may exhibit a heightened susceptibility to calorie-dense but nutrient-poor foods, allowing overweight and obesity risks to accumulate rapidly before traditional micronutrient inadequacies are effectively resolved (38, 39). A parallel shift was observed in gender disparities. Primarily, the advancing age of the cohort participants serves as a fundamental biological factor for the surge in DBM risk among males. Evidence suggests that during the aging process, middle-aged men exhibit a more pronounced susceptibility to skeletal muscle loss and visceral fat accumulation compared to women, a physiological shift corroborated by national reports indicating that growth rates of overweight and obesity among Chinese males have significantly outpaced those of their female counterparts (40), a pattern consistent with our findings in the Guizhou cohort. Within this biological context, the observed lower dietary diversity and simpler dietary structures among males likely exacerbate micronutrient deficiencies, even as the prevalence of overnutrition continues to rise (41). Furthermore, the impact of shifting occupational landscapes has been more substantial for males, characterized by a marked transition from heavy manual labor toward sedentary professional behaviors, which has further suppressed physical activity levels (42, 43), underscoring the impact of evolving occupational structures and gender-specific health behaviors on DBM accumulation. Collectively, these findings indicate that the epicenter of nutritional risk is shifting toward previously lower-risk subgroups, necessitating a proactive recalibration of public health interventions.

Beyond the observed risk reversals across subgroups, the GEE models elucidate several independent contributors to DBM, reflecting its population-averaged effects. Notably, hypertension and high sodium intake emerged as pivotal factors closely associated with increased DBM risk. Our findings indicate that the absence of hypertension is significantly associated with a lower probability of DBM risk, an association that reflects not only clinical co-occurrence but also a profound metabolic interrelationship between these conditions. This interplay is primarily rooted in the imbalance of sodium intake. National surveillance data indicate that sodium consumption among Chinese adults consistently exceeds WHO-recommended thresholds, a surplus that frequently coincides with micronutrient deficiencies (44, 45). In this cohort, hypertension serves as a biological marker of long-term chronic metabolic accumulation, reflecting pathophysiological processes induced by adverse lifestyle and dietary habits. Hypertensive individuals often present with a clustering of overweight, obesity, and other metabolic syndrome (46). Furthermore, emerging evidence suggests that sodium may exert a direct adipogenic effect independent of caloric intake. A dose–response relationship has been observed between salt intake and adiposity, with high-salt consumers facing a significantly higher risk of central obesity. This phenomenon may be attributed to the concurrent consumption of energy-dense processed foods or an increased intake of sugar-sweetened beverages driven by salt-induced thirst (47). The dietary imbalance is equally pronounced at the micronutrient level, where riboflavin exhibits a significant inverse association with DBM risk. Given that over 90% of Chinese adults fail to meet the EAR for riboflavin, a deficiency particularly prevalent among rural residents, who represent the fastest-growing DBM subgroup in this study (48), this finding is highly consequential. It suggests that as dietary patterns shift toward high-sodium, high-energy “Westernized” diets, individuals are increasing their intake of ultra-processed foods while simultaneously reducing the consumption of whole grains, dairy, and green leafy vegetables, the primary sources of riboflavin and other essential vitamins (49, 50).

The protective role of riboflavin against DBM is biologically grounded in integrated metabolic pathways. As a vital precursor to FAD and FMN, riboflavin plays a non-redundant role in mitochondrial energy metabolism and fatty acid *β*-oxidation, thereby modulating energy homeostasis (51). Epidemiological evidence corroborates that riboflavin status is inversely associated with adiposity, whether defined by BMI, waist circumference, or body fat percentage (52). Moreover, inadequate riboflavin intake is linked to elevated hypertension risk and cardiometabolic diseases. This association is particularly salient in individuals with the MTHFR 677TT genotype, a prevalent variant that increases susceptibility to hypertension, where riboflavin supplementation has been shown to mitigate blood pressure by lowering homocysteine levels (53, 54). In conclusion, riboflavin exhibits a potential mitigating role against DBM risk within this population. These insights suggest that optimizing dietary riboflavin intake may offer a promising auxiliary avenue for public health strategies aimed at addressing both micronutrient gaps and metabolic risks. However, given the observational nature of this study, further clinical trials and longitudinal intervention studies are warranted to rigorously evaluate the actual effectiveness of riboflavin supplementation in reversing DBM progression.

To the best of our knowledge, this is the first study to utilize a long-term prospective dynamic cohort to analyze the evolving temporal trends and influencing factors of DBM. Nevertheless, several limitations should be acknowledged. First, nutrient status was inferred from three-day 24-h dietary recalls rather than objective blood biomarkers; this reliance on dietary intake data may introduce information bias, such as inaccurate estimation of portion sizes, and cannot account for individual biological variation in nutrient absorption or metabolism. Second, the assessment of condiment intake was restricted to household consumption, potentially leading to an underestimation of total sodium and oil intake as dining out becomes more prevalent. Third, the lack of seasonal synchronization across survey waves might have introduced seasonal variability in dietary patterns. Fourth, our findings are derived from a single regional surveillance site, which lacks absolute national representativeness. Finally, the potential for residual confounding from unmeasured factors, such as physical activity intensity, cannot be entirely excluded.

In conclusion, our findings underscore a significant shift in the DBM landscape, characterized by the emergence of rural residents and males as new high-risk subgroups. These trends necessitate a proactive recalibration of public health interventions, shifting the focus toward these rapidly transitioning populations to mitigate the escalating crisis of the DBM.

## 5 Conclusion

Over the past three decades, the prevalence of DBM in Southwest China has shown a trend of rapid growth followed by a slowdown in growth, accompanied by a reversal in its urban–rural and gender distribution. These findings underscore the delayed improvement in dietary quality relative to changes in energy structure. Future dietary intervention policies should transition from the traditional “total energy control” approach to a “quality optimization” strategy that prioritizes the correction of micronutrient deficiencies, while promoting increased daily physical activity. Targeted interventions should be developed for emerging high-risk populations, especially rural residents and men, to slow or reverse the development of DBM.

## Data Availability

The datasets presented in this article are not readily available because they contain sensitive participant information and are subject to institutional data sharing restrictions. However, the datasets and analytic code used during the current study are available from the corresponding author (HG) upon reasonable request. Requests to access the datasets should be directed to Hua Guo, guohua_cqy@163.com.
