# Supplementary material for: Longitudinal reversals and evolving patterns of the double burden of malnutrition among adults in Southwest China: a population-based cohort study
Source: Front Nutr. 2026 Jul 8;13:1861218. doi: 10.3389/fnut.2026.1861218 (PMC13389846; doi:10.3389/fnut.2026.1861218)
Supplement: Supplementary file 1 [file Table_1.DOCX]

Supplementary Material

Table S1. Dynamic Distribution of Cross-Sectional Observations and Longitudinal Tracking Status Across 11 Survey Waves (N = 10,703)

| **Survey Wave** | **Total Observations (N)** | **New/Single Enrollment, n (%)** | **Repeated Follow-up, n (%)** |
| --- | --- | --- | --- |
| **1991 (Baseline)** | 1,088 | 1,088 (100.0%) | 0 (0.0%) |
| **1993** | 1,036 | 243 (23.5%) | 793 (76.5%) |
| **1997** | 1,207 | 464 (38.4%) | 743 (61.6%) |
| **2000** | 1,035 | 219 (21.2%) | 816 (78.8%) |
| **2004** | 1,011 | 198 (19.6%) | 813 (80.4%) |
| **2006** | 1,010 | 119 (11.8%) | 891 (88.2%) |
| **2009** | 969 | 101 (10.4%) | 868 (89.6%) |
| **2011** | 956 | 134 (14.0%) | 822 (86.0%) |
| **2015** | 919 | 177 (19.3%) | 742 (80.7%) |
| **2018** | 691 | 103 (14.9%) | 588 (85.1%) |
| **2023** | 781 | 271 (34.7%) | 510 (65.3%) |
| **Total** | **10,703** | **3,117 (29.1%)** | **7,586 (70.9%)** |

Table S2: Distribution of Individual Longitudinal Follow-up Frequency (n = 3,117)

| Longitudinal Attendance Status | Unique Participants, n (%) | Contributed Observations, N (%) | Average Waves per Capita |
| --- | --- | --- | --- |
| Single Attendance  (Only 1 Wave) | 1,118 (35.9%) | 1,118 (10.4%) | 1 |
| Intermittent Attendance  (2–4 Waves) | 1,089 (34.9%) | 2,913 (27.2%) | 2.67 |
| Persistent Attendance  (≥ 5 Waves) | 910 (29.2%) | 6,672 (62.3%) | 7.33 |
| Total Cohort Pool | 3,117 (100.0%) | 10,703 (100.0%) | 3.43 |

Table S3 Multivariable-Adjusted GEE Model

| **Covariate** | **OR (95% CI)** | **P-value** |
| --- | --- | --- |
| **Socio-demographic** |  |  |
| Survey wave (year) | 1.06 (1.05, 1.07) | < 0.001 |
| Age (years) | 1.01 (1.01, 1.01) | < 0.001 |
| Gender (Female vs. Male) | 1.29 (1.08, 1.55) | 0.006 |
| Residence (Urban vs. Rural) | 1.44 (1.23, 1.69) | < 0.001 |
| Education | 1.14 (1.01, 1.29) | 0.04 |
| **Behavioral & Health** |  |  |
| Smoking status (Current vs. Non-current) | 0.94 (0.82, 1.08) | 0.399 |
| Hypertension (No vs. Yes/Medication) | 0.78 (0.71, 0.87) | < 0.001 |
| Chronic diseases (Yes vs. No) | 0.85 (0.61, 1.16) | 0.303 |
| **Dietary Intake** |  |  |
| Total energy intake (per 1 kcal/day increase) | 1.00 (1.00, 1.00) | **0.097** |
| Fat energy ratio (%) | 1.00 (0.99, 1.00) | 0.168 |
| Protein energy ratio (%) | 1.02 (1.00, 1.05) | **0.057** |
| Dietary fiber (g/day) | 1.01 (1.00, 1.02) | **0.04** |
| Cholesterol (mg/day) | 1.00 (1.00, 1.00) | 0.23 |
| Micronutrient deficiency count | 1.01 (0.97, 1.06) | 0.614 |
| Thiamin (Vitamin B1, mg/day) | 0.94 (0.78, 1.13) | 0.478 |
| Riboflavin (Vitamin B2, mg/day) | 0.82 (0.66, 1.03) | **0.087** |
| Niacin (Vitamin B3, mg/day) | 1.01 (0.99, 1.03) | 0.212 |
| Vitamin C (mg/day) | 1.00 (1.00, 1.00) | 0.151 |
| Vitamin E (mg/day) | 1.00 (1.00, 1.01) | **0.047** |
| Potassium (K, mg/day) | 1.00 (1.00, 1.00) | 0.278 |
| Sodium (Na, mg/day) | 1.00 (1.00, 1.00) | **0.047** |
| Calcium (Ca, mg/day) | 1.00 (1.00, 1.00) | 0.365 |
| Magnesium (Mg, mg/day) | 1.00 (1.00, 1.00) | 0.578 |
| Iron (Fe, mg/day) | 1.00 (0.99, 1.01) | 0.204 |
| Manganese (Mn, mg/day) | 0.99 (0.96, 1.02) | 0.411 |
| Zinc (Zn, mg/day) | 1.00 (0.98, 1.03) | 0.774 |
| Copper (Cu, mg/day) | 0.96 (0.91, 1.02) | 0.242 |
| Phosphorus (P, mg/day) | 1.00 (1.00, 1.00) | 0.109 |
| Selenium (Se,μg/day) | 1.00 (1.00, 1.00) | 0.211 |
